# The potential of an online educational platform to contribute to achieving sustainable development goals: a mixed-methods evaluation of the Peoples-uni online platform

**DOI:** 10.1186/s12961-018-0381-2

**Published:** 2018-11-12

**Authors:** Sanjeev Sridharan, Madeleine Bondy, April Nakaima, Richard F. Heller

**Affiliations:** 1 The Evaluation Centre for Complex Health Interventions, St. Michael’s Hospital and University of Toronto, Toronto, Canada; 2 People’s Open Access Education Initiative (Peoples-uni), Manchester, United Kingdom

**Keywords:** Online education, Sustainable Development Goals, evaluation

## Abstract

**Background:**

This paper reports on an online platform, People’s Open Access Education Initiative (Peoples-uni), as a means of enhancing access to master’s level public health education for health professionals. Peoples-uni seeks to improve population health in low- and middle-income countries by building public health capacity through e-learning at very low cost. We report here an evaluation of the Peoples-uni programme, conducted within the context of Sustainable Development Goal 4, which seeks to “*ensure inclusive and quality education for all and promote lifelong learning*” by 2030.

The evaluation seeks to address the following three questions: (1) Did Peoples-uni meet its intended goals? (2) What were the different types of impacts that students experienced? (3) What suggestions for future changes in Peoples-uni did students recommend?

**Methods:**

A mixed methods evaluation consisted of two parts, namely an online survey and a telephone interview. A total of 119 master’s level graduates were invited to participate; responses were obtained from 71 of those invited, giving a response rate of 60%. Respondents were spread across 31 countries. Interviews were conducted with 18 respondents.

**Results:**

There was strong evidence that Peoples-uni had achieved its stated goals. Potential impacts on students included knowledge to enhance practice and appreciation of context, enhanced research capacity through knowledge of public health, critical thinking and evidence-based programming, and empowerment of students about the potential of education as a means of improving their lives. Accreditation through future partnerships with local universities was recommended by students.

**Conclusions:**

Peoples-uni has been able to deliver a credible public health master’s level educational programme, with positive impacts on the students who graduated. Challenges are to find a way to accredit the programme to ensure its sustainability and to see how to take full advantage of the current, and future, graduates to turn this from an education programme into a capacity-building programme with real impact.

## Background

Sustainable Development Goal (SDG) 4 seeks to “*ensure inclusive and quality education for all and promote lifelong learning*” by 2030 [1]. Despite this exciting aspiration, a roadmap by which such a goal can be achieved continues to remain quite unclear. The focus of this paper is an evaluation that explores the role of an online education platform, called the People’s Open Access Education Initiative (Peoples-uni) [2–4], as a means of enhancing access to education. The paper uses the evaluation as a case study to raise questions around what can be learned from the experiences of a number of individuals who completed a Master of Public Health (MPH). This evaluation provides an opportunity to learn from the realities on the ground and also to learn lessons about enhancing access that would be useful in focusing on achieving the aspirations of SDG 4.

An online programme focused on public health is especially relevant, given the focus of a number of SDGs related to health, wellbeing and the social determinants of health. As an example, SDG 1 focuses on poverty, SDG 2 focuses on hunger, SDG 3 on good health and wellbeing, SDG 5 on gender equality, SDG 6 on clean water and sanitation, SDG 10 on reduced inequalities and SDG 11 on sustainable communities. These are all areas that potentially intersect with the focus of a graduate programme in public health.

A number of the SDG goals [1] can potentially be aided by an organisation like Peoples-uni. For example, Peoples-uni can focus on the knowledge and skills needed to promote sustainable development (goal 4.7), continue to refine the online platform to be gender sensitive (goal 4.A), offer scholarships for students in resource-poor settings (goal 4.B), and enhance the supply of qualified teachers through online teaching (goal 4.C).

### Peoples-uni: what it hopes to achieve

Peoples-uni was conceived as a response to the massive health problems facing developing country populations, the need to build capacity to study the causes of and solutions for these problems, and a lack of affordable and accessible educational opportunities that would help build this capacity. The mission of Peoples-uni is “[t]*o contribute to improvements in the health of populations in low- to middle-income countries by building Public Health capacity via e-learning at very low cost*” (http://www.peoples-uni.org/content/overall-objectives). Box 1 describes the objectives of Peoples-uni.

### Brief history

Started in 2007 with registration as a charity in the United Kingdom, the first module was piloted in 2008 and followed by the offering of a few modules later that year. Modules were developed, and then delivered, by teams of volunteers with academic or service experience in public health, using a common structure and populated by Open Educational Resources. The number of modules grew over time, partly by serendipity reflecting the interests of volunteers, and partly by identifying the need to build capacity in the foundation sciences of public health and the problems facing developing country populations. The IT and educational administrative infrastructure also grew over time to meet all the requirements to enrol and support students and volunteer tutors, run modules, assess competence and report results as in a high-quality educational organisation. Students came mainly from Africa, but also from India and other parts of the developing world. Unlike some other capacity-building programmes, for example, the International Clinical Epidemiology Network, where capacity was built within institutions, students enrolled as individuals (although some organisations sponsored some of their staff).

To reflect Objective 3 of the requirement for the education to be at the master’s level, while being classed as a charity rather than a registered provider of higher education, Peoples-uni aimed to enter into partnerships with those who could provide a credible academic award to the students. Manchester Metropolitan University (MMU) in the United Kingdom performed this role for 128 students who enrolled over a 2-year period in a Master of Public Health degree, but following the termination of this partnership, it has proved difficult to identify another academic partner (note: during the time that MMU were validating the programme, some students graduated with an MPH from them, while others graduated from Peoples-uni having taken the same course with the same input and assessment processes. The evaluation we report here includes both groups of graduates).

The use of volunteers, Open Educational Resources and a fully online educational infrastructure allowed courses to be provided at low cost, affordable to most students, yet with a bursary scheme for those unable to pay fees.

### How this differs from Massive Open Online Courses (MOOCs)

The rise of MOOCs occurred at roughly the same time as Peoples-uni was evolving. However, there are a number of differences in the educational approach, including the MOOCs’ heavy reliance on specially created content – mainly videos – rather than the Open Educational Resources used extensively by Peoples-uni. MOOCs do not usually make their resources available for later use by students, while Peoples-uni modules are published under a Creative Commons licence. MOOCs have much less insistence on contributions to discussion forums or tutor facilitation than Peoples-uni, and there are no assignments other than quizzes. Finally, although mini-credits are now being offered by some MOOCs, academic credit is not the general aim.

### Capacity-building for public health: what Peoples-uni hopes to achieve in terms of building capacities

As expressed in Objective 6, Peoples-uni from the start wanted to support its graduates in the role for which they have been trained in the programme, that is, to teach, perform research, and implement evidence-based health policy and advocacy to improve the health of their populations. While the competences gained during the educational programme, and the academic award itself, are important outcomes, the final endpoints of the programme would be the way in which graduates can use their new skills to impact on population health. An active Alumni group has gone some way towards this [5], but Peoples-uni continues to seek mechanisms to fully realise the potential of the programme.

### Brief literature review

In 2007, the date that Peoples-uni was established [3, 4], a paper outlined the extent of the need for education in public health in Africa, stating that “[o]*ver half (55%) of countries do not have any postgraduate public health programme*” [6]. There have been some evaluations of postgraduate public health programmes, including those aimed at low- and middle-income country (LMIC) populations, most of which have reported positive outcomes in terms of skills gained [7–11]*.* We are not aware of evaluations of online master’s level courses in public health aimed at an LMIC audience, although distance learning programmes in Africa have been described [12, 13]. Of relevance to the use of Open Educational Resources (OER), Angell et al. [14] reported on the limited literature on the availability of OER in public health, and subsequently developed a model for the release of OER in public health, which they hoped would “*provide practical assistance and encouragement for the academic public health community to create and share OER*” [15]*.* A thought-provoking paper [16] identifies OER and online learning as “*One of the key concepts in the right to education is access: access to the means to fully develop as human beings as well as access to the means to gain skills, knowledge and credentials….to address the global education gap*”. Addressing the public health global education gap nicely summarises Objective 1 of the Peoples-uni. A fundamental question that this evaluation explores is whether Peoples-uni can help address this gap.

### Evaluation of Peoples-uni

**Fig. 1:**
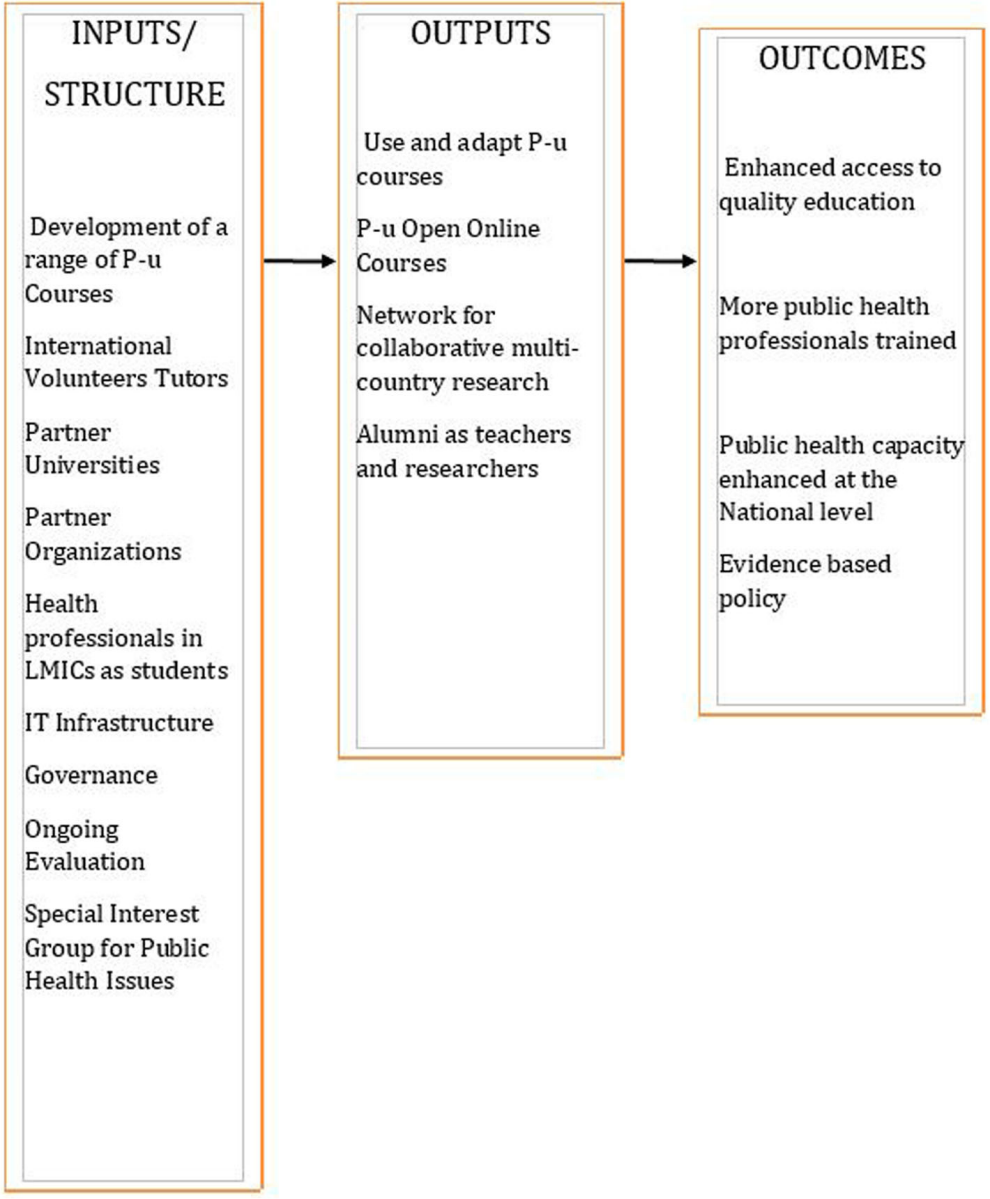
A simplified programme logic model for Peoples-uni (P-u)

The original theory of change for Peoples-uni is described in Fig. 1. Its aspirations of key outputs included collaborative, multi-country research, education using online courses and courses from partners, advocacy efforts, and impacts on evidence-based health policy. Key inputs included development of courses, international volunteer tutors, IT infrastructure, a growing base of alumni, and partnering with universities and other organisations. The tutors would not only teach but would also offer guidance for both research projects and dissertations. Peoples-uni was supported by a structure that included IT infrastructure, governance and a process of ongoing evaluation. The activities were mostly online to enhance collaboration across the course participants.

This theory of change has stood Peoples-uni well over the past decade, but much of the ideas that were generated above were by the planners, who were based in the United Kingdom, Australia and other Western settings. Therefore, there is a need to learn from the students who had successfully graduated from Peoples-uni. It is possible that the impact pathways by which Peoples-uni actually impacted individuals is different from what was originally envisaged. Further, the student participants in the evaluation provided suggestions on how Peoples-uni could be developed in the near future.

The ideas generated by the former students can serve both as a means of planning for future structures and inputs needed to support student needs as well as a process of re-visioning an educational institution like Peoples-uni in light of SDG 4. The evaluation focused on the following three questions:

1. Did Peoples-uni meet its objectives? This aspect of the evaluation is summative, and it seeks to assess experiences and feedback from Peoples-uni graduates against the stated goals of the programme.
2. What are the different types of impacts that the respondents have experienced as a result of Peoples-uni? This question helps us learn from the respondents on an emergent theory of change based on the different contexts of the wide range of Peoples-uni graduates.
3. What are examples of feedback that former students had that can help develop a future version of Peoples-uni? This aspect of the evaluation is formative, and we seek to learn from the former students on how future versions of Peoples-uni can be enhanced.

## Methods

### Data collection

The evaluation was conducted in two parts, an online survey and a telephone interview. In the first part, the students who had graduated with a master’s level award during the time that the course was accredited by MMU were sent an invitation to participate in an anonymous online survey (SurveyMonkey). The invitation was sent by the lead evaluator, deliberately external to the usual online communication system run by Peoples-uni. Questions were based on the objectives of the study, and reflected other evaluations in the literature. As part of the survey, respondents were asked to give their email address if they were prepared to take part in a subsequent interview survey. One reminder was sent to the group after 4 weeks.

From amongst the respondents agreeing to a possible interview, 18 were invited to participate in a semi-structured interview through phone or Skype. Selection was based on the desire to include a spread of participants on the basis of their graduation status (MMU or Peoples-uni) and country. The semi-structured format enabled the evaluation team to set the agenda with respect to the topics covered, and allowed interview responses to determine the kinds of information produced about those topics.

The interview guide was designed by the evaluation team, but reflected questions previously used in the literature [17]. The aim was to capture a rich understanding of the graduates’ interpretations of the Peoples-uni programme. The evaluation team reviewed rapport-building practices to ensure that the interviewer fostered a sense of safety and comfort for the interviewee. All of the interviews were completed over Skype or over the phone.

Each interview was audio-recorded and transcribed in Microsoft Word. Additionally, to ensure confidentiality for the respondents, any identifiers such as names or specific locations were removed from these transcripts before including them in this report and a participant number was assigned to each participant.

### Analytical focus

The three broad evaluation questions described earlier guided the analysis. The summative focus of the analysis assessed progress through analysis of the structured survey against the goals of Peoples-uni. The qualitative interviews provided the data for understanding the impact pathways and formative feedback. As part of the qualitative analysis, two evaluators sought to identify key categories of impacts and feedback from the respondents. The analysis was informed by an understanding of the existing theory of change as well as recognition that the feedback helped to inform a refined theory of change.

Data were analysed using thematic content analysis, the most common method of qualitative analysis, which is both grounded in the literature and consistent with an interpretive theoretical paradigm. This study applied an inductive style of analysis to derive themes and explanations from a close reading of the data, rather than through an attempt to categorise the data into pre-existing concepts or ideas from theory. To explore graduates of the Peoples-uni programme’s interpretations and familiarise themselves with the data, two research team members re-read each transcript and its corresponding memo.

### Ethical issues

As part of the application process for entry to Peoples-uni courses, students are informed that their anonymised information might be used for research into the outcomes of the education programme and are provided the opportunity to reject this. The invitation letter to the online survey made it clear that this was carried out by an independent organisation, and that participation was voluntary and anonymous. Those agreeing to be interviewed had to provide their names and email addresses.

For the interview phase, each participant was informed of the interview subject matter and was debriefed about the evaluation purposes afterwards. Prior to commencing their interview, each interviewer emphasised voluntary participation, and the right to confidentiality. Special care was taken to maintain respect and confidentiality of the participant and their information. Given these precautions, there were no anticipated harms from this evaluation.

## Results

### Online survey

#### Respondent background

A total of 119 students who had joined the Peoples-uni programme and graduated at the master’s level were invited to participate in January 2017, 89 of whom had gained their award from MMU and 30 gained their award from Peoples-uni alone. Responses were obtained from 71 of those invited (60%); 45 said they were MMU graduates (51% response rate) and 22 said they had the Peoples-uni qualification alone (73% response rate) (4 did not answer).

Respondents came from 31 countries. Table 1 shows the major geographic regions from which they came for those invited, those who responded and those subsequently interviewed. The majority came from Western and Eastern Africa, with Southern Africa and the Indian sub-continent making up the other main groups.

**Table 1:** Geographic distribution of survey participants

Region	Invited to survey	Responded to survey^a^	Interviewed
Western Africa	35	14	6
Eastern Africa	33	20	4
Southern Africa	18	11	4
Indian sub-continent	15	9	2
Elsewhere	18	12	2
Total	119	66	18

^a^ Five respondents did not give this information

The respondents come from a wide variety of roles and positions in public and private healthcare, including medical practitioners, managers, project coordinators, academics and researchers, working in a variety of settings from community-based NGOs to regional or global organisations.

We were able to examine the date of, and age at, graduation as well as gender for all 119 of those who were invited to participate (including respondents who offered to be interviewed and hence gave their names, as well as those who were interviewed); 33 (28%) had graduated in 2012 or 2013, 59 (50%) in 2014 or 2015, and 27 (53%) in 2016 or 2017. Among the whole group, 58 (50% of 116 with data) were aged under 40 and 13 (11%) were aged 50 or more. There were only small differences in date and age at graduation between the overall group, those agreeing to be interviewed and those who were actually interviewed. In the total Peoples-uni experience, 37% of the students were female (Heller RF, Strobl J, Madhok R: Online education for public health capacity building in low- to middle-income countries: the Peoples-uni experience. The International Review of Research in Open and Distributed Learning, forthcoming); among the 119 graduates surveyed for this study, there were 37 (31%) females, 12 (23%) of the 53 who agreed to be interviewed were female, and only 1 (6%) of the 18 actually interviewed was female.

#### Impacts of Peoples-uni

Overall, 57% (35 of 61) of respondents said they would not have been able to get an MPH without attending Peoples-uni. The main goals that participants hoped to achieve by enrolling in the MPH programme were to advance/upgrade their public health knowledge and skills and to obtain a degree from an accredited institution. The other main goals that students had were to advance their careers and improve their research knowledge. In total, 94% (62 of 66) of respondents agreed that their expectations of Peoples-uni were met.

In response to the question “What knowledge and skills have you acquired in the MPH programme that have had an impact on your professional practice, if any?”, the skills that were most frequently reported were research skills, epidemiology and biostatistics skills, programme design and evaluation skills, evidence synthesis skills, and health promotion skills. A number of other skills were also mentioned.

Of 61 respondents who answered the question, 35 (57%) stated that their employer valued the new skills that they acquired through the MPH, 6 (10%) that it was not valued, while 20 (32%) were not sure. Respondents who stated that their employer valued their new skills provided examples of how they were given a promotion and excellent performance appraisals as well as more responsibility overall and opportunities to teach and be involved in research. Those who felt that their employer did not value their MPH degree cited reasons such as their employer not believing in online education or that formal recognition was missing.

Among 61 respondents, 25 (41%) had a promotion, new position or salary increase as a result of their participation in the MPH programme. Graduates were given a variety of promotions or new positions such as managerial or specialist roles.

In total, 85% (57 of 67) of respondents stated that the programme contributed to their research in a practical way, most attributing this to the MPH programme. Examples included the publication of at least one paper (*n* = 12), receiving funding for at least one project (*n* = 4), supervising at least one other person’s research (*n* = 11), and undertaking or participating in research (*n* = 31), all since gaining their MPH.

Additionally, 15 respondents had already enrolled in a PhD programme since graduating, the majority in public health or global public health. A further 43 respondents stated that they planned to enrol in a PhD programme. Participants who were not yet enrolled stated that they were looking for an opportunity to apply, were facing barriers to apply because of resources and accessibility concerns, or could not apply because their MPH is not recognised.

A total of 51 of 61 respondents (84%) were already teaching the skills they had gained to others either as tutors or Student Support Officers within Peoples-uni courses (*n* = 12), on local programmes or courses (*n* = 11), and to their local colleagues (*n* = 28). Overall, 60 of 62 respondents (97%) felt that they had been provided with the skills to implement local public health programmes by Peoples-uni, giving many detailed examples, and all of the 61 who answered felt that the course content was applicable to the context of public health in their own country.

### Interview survey

#### Impact pathways

Box 2 describes some of the feedback from respondents corresponding to each of the impact pathway categories that emerged from analysis of the interviews, which included:

1. *Enhanced practice and appreciation of context:* A number of the respondents described how taking courses from Peoples-uni helped them enhance their practice. As an example, it helped some of them to think about the population health aspects of their actions and interventions. A number of respondents suggested that Peoples-uni courses had helped them incorporate considerations of context into their practice both from theoretical and scientific perspectives.
2. *Enhanced research capacities:* One example of impact that was mentioned by a number of the respondents was greater appreciation of the different types of research, models and theories, with a specific enhancement of knowledge of public health, critical thinking skills and evidence-based programming.
3. *Empowering individuals through education:* A few of the respondents spoke about how Peoples-uni helped empower them to think about the role of education to improve their own lives.
4. *Visioning a critical mass for national-level impacts:* For some of the participants, there was also recognition that an institution like Peoples-uni can help build capacities at the national level if there is a critical mass of individuals locally who had also received training from Peoples-uni.
5. *Building confidence to enrol in a doctoral programme:* The programme also helped encourage a number of students successfully to enrol in a doctoral programme.
6. *Mentoring opportunities:* A few of the respondents also spoke about the value of the mentoring programme and how such opportunities provided by Peoples-uni helped raise both confidence and knowledge.
7. *Career enhancement:* A number of the respondents mentioned that obtaining an accredited degree from Peoples-uni also helped them get jobs or promotions at work.

#### Process feedback

The process feedback is summarised below. Box 3 describes some examples of feedback received from the respondent for each of the feedback themes. Key areas of feedback included:

1. *The need for accreditation:* An overwhelming majority of the respondents felt that it was vital for Peoples-uni to be accredited. The current lack of academic accreditation is the key threat to the sustainability of the programme, and was clearly and passionately articulated by nearly every respondent.
2. *Accreditation through local universities:* A number of students also provided feedback that accreditation could happen through local universities. A few of the students themselves suggested a role that they could play in obtaining such accreditation through local universities.
3. *Expanding the role of the alumni:* A number of suggestions were received highlighting the role that the alumni could play in future educational efforts. Examples of feedback included suggestions for support for alumni in grant proposal writing and a process by which alumni could provide support for new students.
4. *The need for more stringent quality standards:* A few respondents also suggested that there needed to be a clearer set of standards and better-quality filters to ensure that students meet standards before joining Peoples-uni (of note, criteria for entry to the Peoples-uni master’s programme is solely a pass in two modules at the master’s level – this being considered evidence of adequate academic and language skills to navigate a course at this level).
5. *Providing transcripts:* A few students reported difficulties getting transcripts from Peoples-uni.
6. *Further incorporating local context:* Feedback was received stating that a future version of Peoples-uni could incorporate local context issues even further. Some of this feedback focused on courses that could additionally incorporate local contexts; other feedback suggested the potential of building partnerships with local health systems.
7. *Raising salience of Peoples-uni through collaborations:* There were also a number of suggestions for building collaborations that could help enhance the students’ achievement of getting a MPH. The insight in this suggestion was that the added value of Peoples-uni did not have to happen just through accreditation but could be established through other pathways, including collaborations with local health system partners and other academic institutions like the Royal Society for Public Health.
8. *Future courses:* A number of ideas were received for future courses that needed to be offered. These included courses on motor traffic injuries, occupational health, project management, non-communicable diseases, health informatics and ethics.
9. *The need for sponsors:* A few respondents suggested the need for Peoples-uni to look for sponsors who can help with funding students.
10. *Technology to enhance Internet access:* Some suggestions were received on enhancing technology for delivering courses to allow students to access courses even in settings where Internet access is hard.
11. *Building awareness of Peoples-uni:* A few respondents suggested that more could be done to improve the awareness of what Peoples-uni offers.
12. *Improving follow-up after the dissertation:* Another set of feedback focused on improving the follow-up supports after the completion of the dissertation. Such supports could help students raise their visibility through publications or presentations in conferences.
13. *The need for a physical presence*: Further feedback suggested the physical presence to provide opportunities for students to interact in person.

## Discussion

This paper discusses, using a case study of Peoples-uni, the potential role that online education can play in achieving SDG 4. One remarkable aspect of Peoples-uni was its ability to reach individuals within a wide variety of countries. This in itself speaks to the potential of an online environment as a tool for achieving SDG 4.

This evaluation has demonstrated that an online platform focused on public health, like Peoples-uni, can result in multiple types of impacts. This evaluation helps generate learning about the impact pathways by which an online educational institution might work. Potential pathways included developing knowledge to enhance practice and appreciation of context; enhanced research capacity through knowledge of public health and evidence-based programming; empowering individuals about the potential of education as a means of improving their lives; developing a vision of the critical mass needed of individual capacities for national level impacts; building confidence, skills and capacities of students to enrol in doctoral programmes, in part through providing mentoring opportunities; and enhancing career prospects by obtaining an accredited degree. Although the goal of this evaluation was to help the Peoples-uni planners and implementers to rethink and refine their theory of change, the major message has been to confirm the theory of change, as set out in Fig. 1. The main reflection is to emphasise the importance placed by the students on the ‘input’ section of the theory on the need for university partnerships to provide a credible academic award for career development.

Despite this evaluation focusing on the graduates of Peoples-uni, we feel that some of the lessons will be of broader relevance to the field of health and beyond. A volunteer-led organisation outside the traditional higher education system has been able to create an international volunteer tutor workforce and use the benefits of OER and an open educational platform to create an educational programme valued by its graduates in LMICs. The products of this and similar programmes are likely to be of value to global interventions such as SDGs. Evaluations like this will be of use in guiding future refinements within programmes such as this one.

This evaluation has also generated ‘learnings’ on how an online education platform like Peoples-uni can be enhanced to better address the constraints and barriers of students working and living in settings that can, at times, be both resource poor and poor in internet connectivity. Key process learnings from the evaluation included the need for Peoples-uni to partner with local universities in order to obtain accreditation; the need for providing additional support for students after their dissertation; having more stringent criteria for admissions; Peoples-uni providing better follow-up support, including providing transcripts (online transcripts are currently available to download, but are not paper based); and, if possible, having more of a physical presence, including providing opportunities for students to attend local workshops and conferences, most likely in African countries. Most of the above points would require more capacity than Peoples-uni presently has available. In order to address the capacity gap, we think an organisation like Peoples-uni needs to develop strong partnerships, both globally and regionally.

The impact pathways identified by the graduates are not dissimilar to those that may be expected from any successful educational programme. A further impact that might have been identified by the graduates from our online programme would be the commitment to volunteerism, since the tutors are all volunteers. This is actually demonstrated by the finding that a number of graduates have themselves joined Peoples-uni as volunteer tutors and are passing their skills to others in this and other ways. In a separate but related theme, the use of OER as the main source of the resources used in the programme encourages the sharing of educational materials for the common good. The theme continues into the demonstrated importance of collaboration – the online format has discussion forums that encourage collaboration among students and among the tutors (each module having at least five tutors to spread the load). The whole programme is a demonstration of international collaboration, with tutors from more than 50 countries and students from nearly 100 countries. Further, its impact is demonstrated by the number of published papers resulting from collaboration among alumni [5, 18, 19]. Other strengths of the online format in general are the ability to combine study with work and family life, and for those geographically remote from a university campus. More than half of the graduates said they would not have been able to gain an MPH in any other way.

### Were the objectives of Peoples-uni met?

It is too early to say if the first part of the mission of Peoples-uni, namely “[t]*o contribute to improvements in the health of populations in low- to middle-income countries*”, has been met, but the programme has clearly met the remainder “*by building public health capacity via e-learning at very low cost*” (http://www.peoples-uni.org/content/overall-objectives).

The programme has provided public health education, with the majority of graduates coming from Africa or the Indian sub-continent, and including a wide range of types of health professionals. Many said that they would not have been able to get an MPH without Peoples-uni. Further, it was encouraging to see that such a high proportion of the graduates were already passing their training on to others, and the ‘train the trainers’ objective was being met.

Although career development of the students was not specified as an objective of the programme, many of the questions in both parts of the evaluation cover this aspect. Career advancement, such as promotion, new positions or responsibilities, appreciation by the employer, as well as accessing the next educational step of a PhD, appear to have been welcome and frequent outcomes of the programme. Critical thinking was one of the interview themes that emerged, and is found as an underlying theme of much of the educational programme. It was also encouraging that such a high proportion of the respondents felt that the programme fits them to deal with local public health problems. The high proportion of respondents who had already published papers or started research is also a positive outcome of relevance to improving the health of their populations.

The existence of a growing cohort of well-trained public health professionals, who are already maintaining collaboration in education and research [5], is a resource to be nurtured and expanded. It is to be hoped that, in a next phase of the activities of Peoples-uni, a way to utilise this resource to further meet the objective of improving the health of populations will be found.

### Building capacities for global health action: the salience of Peoples-uni at a time of SDGs

The evaluation also provided former students the opportunity to offer input to enhance future versions of Peoples-uni. Clearly, the leading challenge raised by most students was the need for accreditation. Getting accreditation from universities that are based in countries like the United Kingdom, Australia or New Zealand has proved to be a serious challenge. Consistent feedback from a number of students was to seek out accreditation from local universities. While we think that this is an attractive way forward, it also raises a number of challenging questions, such as (1) What are the incentives for local universities to collaborate with an online platform like Peoples-uni? (2) What are the mechanisms by which such partnerships can both help the quality of public health training in local universities without having negative economic impacts for the university? (3) What role should international organisations and funders like the UN or WHO or global foundations play in promoting such partnerships between an online platform and local universities? (4) Can action-focused partnerships be created between international organisations, online platforms and local Universities not only for accreditation but also to build capacities on the ground for action?

The tropEd Network for International Health in Higher Education is a good example of networked partnerships between universities, and has emphasised the importance of quality assurance in transnational higher education [20]. Other than partnering to provide an educational programme, forms of partnership to capitalise on the ‘products’ of educational programmes in collaborative research, evaluation and policy will require a different set of partners and models. Local, national and international health service providers now become possible partners. As described below, these will have the potential to help meet the SDG targets. It is important to remember that benefits for each member of a partnership is an essential component for success and sustainability (of note, since the paper was submitted for publication, Peoples-uni has established a partnership with Euclid University (www.euclid.int), which will offer an MPH to Peoples-uni graduates).

We believe that answering the above questions will be critical, not just for Peoples-uni but also to move towards more thoughtful models of capacity-building that pay attention to the inequities in access to education that exist between different countries. As we work towards achieving the SDGs, what role should accessible education through a platform such as Peoples-uni play in building capacities in settings where existing training in public health is weak? Could an online platform like Peoples-uni have a role to play in disrupting existing inequities in educational systems?

The feedback from multiple students clearly generated data that provided evidence that an online platform can help enhance individual-level access. Yet, Peoples-uni or online platforms are unlikely to be disruptors of existing orders without active partnership either at the local or national levels or partnerships with organisations like the UN, WHO or international foundations. One of the great strengths of Peoples-uni is that established networks of alumni are already working in settings that may have an important role in reducing inequalities in health and education.

### Towards global health action

Key actions that emerge from the discussion are as follows:

1. There needs to be dialogue around how an educational organisation like Peoples-uni can get help in accreditation. Given SDG 4’s focus on reducing inequities in education, such a dialogue becomes critical.
2. There need to be efforts to build such accreditation through partnerships with local universities based in country settings. We think organisations like the UN or foundations with global reach have a role to play in building such partnerships.
3. Both of the above dialogues around accreditation and local partnerships need to be accompanied by a focus on the types of quality, with an eye on the excellence or quality needed to achieve the multiple SDG goals.
4. It is also worthwhile to have a broader discussion around the public health capacities that are needed globally, nationally and locally to address the SDGs. An important question that emerges from our work here is the important role that online institutions can play in enhancing access. Going forward, one way to conceptualise the needs of building capacities for addressing SDGs is to think about a distributed network of capacities that are available globally, and especially locally, based on need (by distributed network we imagine one centralised quality assurance and implementation group that also includes a number of national and regional partners that are also leading in implementation and incorporating contextual knowledge). Low-cost platforms with a far reach have a role to play in further developing such a distributed network. The distributed network will need to be sensitive of the heterogeneities of problems/needs across and within different countries/regions and also the varieties of capacities that already exist.
5. Based on the feedback, such a distributed network would include courses that pay attention to the varieties of local contexts that public health practitioners work in. We believe knowledge of such local contexts will be increasingly important in addressing the SDGs. We also think that a critical mass of trained public health professionals within a nation or a region who understand the basics of public health as well as evidence-based programming provides opportunities to conceptualise capacity-building at the country or perhaps even the regional level.

### Study limitations

Although the evaluation was conducted externally in the hope that this would reduce any tendency to want to post only positive responses, we are not able to discount the possibility of response bias. Non-respondents might well have been less positive about the stated value of the programme. The response to the questionnaire part of the study was greater among those who said had gained their award from Peoples-uni than those whose award came from MMU, and this may have been because they were keen to reflect their views of the importance of the need for future university partnerships to improve the credibility of the award – a key and consistent finding of the study. In addition, we did not validate or triangulate the answers from other sources, such as by interviewing employers. Further, those interviewed were not representative of all graduates in terms of gender due to the selection criteria being restricted to geography and the body giving the master’s award. This was not an intended consequence, and may have biased the interview responses in an unknown direction.

The first of our study questions of whether Peoples-uni met its objectives, which include improvements to the health of populations, can only partly be answered by a survey of graduate experience and opinions. It is possible that the positive career developments described by the graduates might have occurred without their enrolment in Peoples-uni. It was also probably too early in the life history of the graduates to explore the wider impacts on society or the workplace. As part of the dissertation assessment, students are asked to reflect on the impact that the course has had on their work, and there are many anecdotal reports of such impact.

Finally, by focusing on the graduates of the programme, we appreciate that these represent the success stories. We are not able to capture the reasons why these people actually managed to enrol and then succeed while others who might have benefited missed out. A future evaluation might tackle this issue and help identify ways to open up education further via the Peoples-uni approach, especially for women.

## Conclusion

It appears that, as with other evaluations in the literature, Peoples-uni has been able to deliver a credible public health master’s level educational programme with positive impacts on the students who graduated. The online and low-cost nature of the programme due to the use of volunteers and OER has allowed students to avail themselves of the programme. Graduates are well placed to contribute to improvements in the health of their populations through education and research. The two main challenges are now to first find a way to accredit the programme to ensure its sustainability, and second, to see how to take full advantage of the current, and future, graduates to turn this from an educational into a real capacity-building programme with real impact. The online nature of the programme provides the possibilities of scale and reach, and future partnerships will allow the original mission of Peoples-uni to be met.

## Box 1 Objectives of Peoples-uni

1. Provide public health education for those working in low- and middle-income countries who would otherwise not be able to access such education, via Internet based e-learning

2. Utilise a ‘social model’ of capacity-building, with volunteer academic and support staff and Open Educational Resources available through the Internet, using a collaborative approach and modern information and communication technology

3. Offer education at the ‘train the trainers’ benchmark and master’s degree level for those with prior educational and occupational experience

4. Provide education that meets identified competences which help with the evidence-based practice of public health and are action oriented, to assist in tackling major health problems facing the populations in which the students work

5. Create an educational portfolio including continuing professional development modules and awards of Certificate, Diploma and Master of Public Health

6. Work with the graduates of the educational programme, and other relevant partner organisations, in teaching, research, implementation of evidence-based health policy and advocacy to improve the health of their populations

## Box 2 Examples of self-reported impacts

### Enhanced practice and appreciation of context

“*PU has built my interest in managing healthcare instead of individual patient management. I was reluctant to think outside of an individual patient. When the MOH* [Ministry of Health] *asked me to join him in his team I was reluctant. I thought I was a good clinician but not that I could join public health. Health economics and health equity helped me to decide to join the Ministry. It took me two years to decide to join the MOH. But for PU* [Peoples-uni] *I would not have joined the MOH. One of the arguments from the Minister was that, ‘You are managing individuals. You get frustrated when there is no medicine, a patient is poor etc.’ That is what PU taught me*.” Respondent 15

“*It has also helped to contextualise what I do on the field and bring in academic or theoretical ground to what I do. I now know that behind the implementation there was a science.*” Respondent 14

### Enhanced research capacities

“*PU* [Peoples-uni] *has helped me a lot. I was calling myself a public health professional before PU. After going through it I realised I was not a fully-fledged public health professional. I am able to know how to do certain things like epidemiology. There are a lot of things that were brought by PU which made me a very effective public health person. I now have a number of friends who are asking me for help on how to write a dissertation. I am a mentor*.” Respondent 4

### Empowering individuals through furthering education

“*PU* [Peoples-uni] *is one of the initiatives which is very innovative in the sense that it is a programme which is trying to empower people from low-income countries like my country in the third world. People who cannot afford higher education. It helps a lot. Despite the fact that most of the people here are unable to acquire a degree. The affordability itself helps people to advance themselves. It helps people to expound their scope of knowledge and as a result become more effective because of the knowledge that they gain from PU. In this case PU has been an eye opener for most of us …*.” Respondent 4

### Visioning a critical mass for national-level impacts

“*People are doing a great job at the uni. As of now there are less than 5 alumni in Malawi. Three of us started with it. I understand that some others are students from Malawi that have benefited from PU* [Peoples-uni]*. If there were more of us, the impact from PU would be felt country wide. More and more students would come on board overtime. We did not know about PU initially. The impacts are not yet felt in Malawi because not many people know about it. As the time goes on they will. The job itself that PU is doing is very good. It has to be incorporated for many people*.” Respondent 4

Building confidence to enrol in a doctoral programme

“*I can tell you that after I graduated, I have now registered for a PhD. I have already done 5 courses and I have been performing well. I have a very good foundation from People’s Uni* [PU]*. I know 80% in the courses (biostatistics, epi) PU has done a great job because if you didn’t do your MPH very well you cannot perform at the PhD level. I encourage them to continue*.” Respondent 2

### Mentoring opportunities

“*PU* [Peoples-uni] *has been very helpful to me in a number of ways. First it has provided mentorship. Because I am a tutor – I have gained skills and knowledge on how to deliver something to a mentee…*” Respondent 1

### Career enhancement

“*It has helped me. One of the examples that I can relate to is around the time I was offered a programme position. Prior to that time, I was working in clinical research. I would go out into the field and work with people at a community and population level. It was right off the bat. The skills from the Master’s programme were what I utilised. I was offered the position because I had an MPH degree. This is an example of how the master’s degree was beneficial to me at the time. I needed to have a background in public health. The MPH was handy as it boosted my CV and allowed me to get into the PhD programme*.” Respondent 12

## Box 3 Examples of process feedback

### Process feedback on accreditation

“*It is very important to have the accreditation. In Africa, we are not used to online studies and you must submit your certificate. It is better to come from a strong accredited university like MMU* [Manchester Metropolitan University]*. It helps you to be accepted*.” Respondent 1

### Process feedback on accreditation through local universities

“*The accreditation could be given by local universities. I would be able to help build those connections in Ethiopia that is my job*.” Respondent 15

### Expanding the role of the alumni

“*From my personal opinion. I think there are two ways in which they could better support students. One is to help alumni volunteer in a number of ways. They already have that in place. There should also be more research collaborations. Alumni have emphasised a number of papers and opportunities. Moving forward, part of what would be beneficial for someone would be more academic work, writing papers and all of that. If there would be more opportunities for more collaborations and publishing it that would go a long way*.” Respondent 12

### The need for more stringent quality standards

“*One thing that PU* [Peoples-uni] *needs to do is check the background of the people who apply and see if they are qualified. Secondly, I was thinking in terms of a supervisor, they take a passive role. We need supervisors who are more active in their role. As time moves on they start phasing out, maybe they have too many roles. In my experience my supervisor initially contributed a lot but less and less over time. We need PU to engage more with supervisors and see more if they can engage with students*.” Respondent 10

### Providing transcripts

“*One problem that I had was that I wanted to have the degree evaluated for employment. PU* [Peoples-uni] *wouldn’t give me a transcript. They should also be flexible. Open new doors and ideas. I felt left out. They weren’t interested in submitting the transcript. I felt left out and they should take input from the alumni and help them to be supported. Give transcripts and certificates. Get alumni input and ideas – that would be most important. In the future they could better create something like this*.” Respondent 6

### Incorporating local context

“*I think that PU is doing a good job of addressing the local public health issues. Healthcare providers need to appreciate epidemics. One of the strengths of the programme is this focus – particularly with communicable diseases. One recommendation could be to put epidemics in the local context. Currently, they are mostly generic and used as a case example. They could make it something that would be tangible and that people from anywhere could use*.” Respondent 14

### Raising salience of Peoples-uni through collaborations

“*Another thing is that I have a fellowship with the Royal Society for Public Health* [fellowship]*. That kind of membership overshadows the need for accreditation. I was offered a job. The qualification helped me to get a job. PU* [Peoples-uni] *should think about further collaborations like this*.” Respondent 6

### The need for sponsors

“*The other thing is it has to continue with the issue of looking for sponsors who can sponsor students who have the potential but do not have the money to do the education. If they can look for some more sponsors that would be helpful. The initiative itself is very*.” Respondent 4

### Technology to enhance internet access

“*Another thing is the technology that you are using. They should find a way you can get access to the courses even when the internet is not very good*.” Respondent 2

### Building awareness of Peoples-uni

“*I am looking forward to see if PU* [Peoples-uni] *can improve their advertising so that more people can be aware of what it is that they do. I think we also need to talk to the institutions so that we can understand what it is that PU offers. In Malawi it is very difficult to get Internet, you have to be in town. If there was some way that if you were going around the schools like maybe the secondary schools it would be helpful for people to know what PU offers. It would be quite good for people to know. For advertisements we need local organisations involved to share the word about PU*.” Respondent 8

### Improving follow-up after the dissertation

“*I think one of the things that should be improved upon should be the dissertation. They should emphasise that students should publish work. Even if it is just bringing it to a conference. Some organisations make their own research journals, etc. they should make it open for the academic world to see their work and support it*.” Respondent 12

### The need for a physical presence

“*One more idea would be seminars, workshops, conferences that PU* [Peoples-uni] *can expand. This does not have to be online. PU can organise public health seminars in different locations where students are represented. All over the world PU can organise workshops and conferences. This would provide opportunities for students to publish their work and share what they learned. Students can come and present. That is something that can be looked upon. Something for a future plan.*” Respondent 12
